# Tuberculous Disease of the Bladder

**Published:** 1898-05-21

**Authors:** 


### TUBERCULOUS DISEASE OF THE BLADDER.

Tuberculous disease of the bladder is a malady the treatment of which is peculiarly unsatisfactory, not merely from the fact that it is often secondary to similar disease in other parts, but because, even if primary, it is only occasionally that it is possible to place the patient in the best position to obtain relief from constitutional treatment, while local applications are of very limited utility. A very interesting discussion on the subject took place at a recent meeting of the Medical Society, when Dr. Mansell Moullin urged that an attempt should be made to relieve and, perhaps, even cure such cases by arriving at a diagnosis and undertaking treatment at a much earlier date than was usually done. What he pointed out was that the bladder rarely became infected through its epithelial surface. Nearly always the bacilli invaded it either by direct continuity from some neighbouring organ or through the medium of the blood-vessels or lymphatics. Thus it happened that tuberculous cystitis, if not secondary to pyelitis, was often primary, and if diagnosed early, as ought always to be possible now that the cystoscope and centrifugalisation of the urine were in common use, such disease might often be thoroughly eradicated by suprapubic cystotomy, followed by excision or erasion of the growth. Everything would seem to hinge upon early diagnosis. It is no use attempting a radical cure when the bladder has become so rigid and contracted that suprapubic cystotomy is itself almost out of the question; but Mr. Mansell Moullin pointed out that if the diagnosis was made at the beginning, as it should be, excision or erasion of the growth offered a fair prospect of cure in cases in which the infection of the bladder was primary, and that even in those cases in which other organs were involved as well, the operation would, with very little risk to life, provide a means by which much of the suffering commonly caused by the disease might be prevented.

